# Study of a SARS-CoV-2 Outbreak in a Belgian Military Education and Training Center in Maradi, Niger

**DOI:** 10.3390/v12090949

**Published:** 2020-08-27

**Authors:** Jean-Paul Pirnay, Philippe Selhorst, Christel Cochez, Mauro Petrillo, Vincent Claes, Yolien Van der Beken, Gilbert Verbeken, Julie Degueldre, France T’Sas, Guy Van den Eede, Wouter Weuts, Cedric Smets, Jan Mertens, Philippe Geeraerts, Kevin K. Ariën, Pierre Neirinckx, Patrick Soentjens

**Affiliations:** 1 Laboratory for Molecular and Cellular Technology, Queen Astrid Military Hospital, 1120 Brussels, Belgium; christel.cochez@mil.be (C.C.); gilbert.verbeken@mil.be (G.V.); 2 Unit of Virology, Department of Biomedical Sciences, Institute of Tropical Medicine, 2000 Antwerp, Belgium; pselhorst@itg.be; 3 European Commission, Directorate-General Joint Research Centre (JRC), 1050 Brussels, Belgium; mauro.petrillo@ec.europa.eu (M.P.); guy.van-den-eede@ec.europa.eu (G.V.d.E.); 4 Clinical Laboratory, Queen Astrid Military Hospital, 1120 Brussels, Belgium; vincent.claes@mil.be (V.C.); yolien.vanderbeken@mil.be (Y.V.d.B.); julie.degueldre@mil.be (J.D.); france.tsas@mil.be (F.T.); 5 Queen Astrid Military Hospital, 1120 Brussels, Belgium; wouter.weuts@mil.be; 6 14th Medical Battalion, 1800 Peutie, Belgium; cedric.smets@mil.be; 7 Medical Component, Ministry of Defense, 1140 Brussels, Belgium; jan.mertens@mil.be (J.M.); philippe.geeraerts@mil.be (P.G.); pierre.neirinckx@mil.be (P.N.); 8 Department of Biomedical Sciences, Institute of Tropical Medicine, 2000 Antwerp, Belgium; karien@itg.be; 9 Department of Biomedical Sciences, University of Antwerp, 2610 Antwerp, Belgium; 10 Center for Infectious Diseases, Queen Astrid Military Hospital, 1120 Brussels, Belgium; patrick.soentjens@mil.be; 11 Department of Clinical Sciences, Institute of Tropical Medicine, 2000 Antwerp, Belgium

**Keywords:** SARS-CoV-2, coronavirus, COVID-19, quantitative RT-PCR, serology, military, outbreak, genomic epidemiology, Niger

## Abstract

Coronavirus disease 2019 (COVID-19) caused by severe acute respiratory syndrome coronavirus 2 (SARS-CoV-2) compromises the ability of military forces to fulfill missions. At the beginning of May 2020, 22 out of 70 Belgian soldiers deployed to a military education and training center in Maradi, Niger, developed mild COVID-19 compatible symptoms. Immediately upon their return to Belgium, and two weeks later, all seventy soldiers were tested for SARS-CoV-2 RNA (RT-qPCR) and antibodies (two immunoassays). Nine soldiers had at least one positive COVID-19 diagnostic test result. Five of them exhibited COVID-19 symptoms (mainly anosmia, ageusia, and fever), while four were asymptomatic. In four soldiers, SARS-CoV-2 viral load was detected and the genomes were sequenced. Conventional and genomic epidemiological data suggest that these genomes have an African most recent common ancestor and that the Belgian military service men were infected through contact with locals. The medical military command implemented testing of all Belgian soldiers for SARS-CoV-2 viral load and antibodies, two to three days before their departure on a mission abroad or on the high seas, and for specific missions immediately upon their return in Belgium. Some military operational settings (e.g., training camps in austere environments and ships) were also equipped with mobile infectious disease (COVID-19) testing capacity.

## 1. Introduction

Coronavirus disease 2019 (COVID-19) is an infectious disease caused by the newly emerged severe acute respiratory syndrome coronavirus 2 (SARS-CoV-2), a single stranded-stranded RNA beta-coronavirus with a 29,903 nucleotides-long genome [1]. It was first identified in January 2020 in Wuhan, China, and spread rapidly causing an ongoing worldwide pandemic. The Republic of the Niger is the largest country in West Africa, landlocked by Libya, Chad, Nigeria, Benin, Mali, Burkina Faso, and Algeria. It has a population of about 22 million, living mostly in clusters in the far south and west of the country. Niger faces serious development challenges due to its desert terrain and overpopulation, but also due to attacks by “Al-Qaeda in the Islamic Maghreb”, its use as a transit country for migrants, and the spillover of Nigeria’s Boko Haram insurgency into southeastern Niger. French, American, and Belgian armed forces are currently assisting Niger in countering jihadist groups present in the Sahel [2]. Since the end of August 2019, Belgian military personnel have been deployed in the Maradi region nearby the southern border with Nigeria in the context of Operation New Nero (ONN). The Belgian military are based on the outskirts of the city of Maradi, the second- or third-largest city in the country. In 2012, it had about 267,000 inhabitants and it is one of Niger’s most economically prosperous cities and the major transport, trade, and agricultural hub of South-Central Niger.

A group of 70 Belgian soldiers was sent in two waves, one on 12 December 2019 and the other on 1 February 2020, to the Maradi camp. They formed the “Mobile Education and Training Team” (METT), which brings together 12 military trainers, members of the Special Forces group, around forty members of the 3rd Battalion Para and a “Special Operations Surgical Team”. They trained a special intervention company made up of 150 Nigerien soldiers.

The first case of COVID-19 in Belgium was confirmed on 3 February 2020 [3]. On 19 March, the first case of COVID-19 in Niger was reported in the Nigerien capital, Niamey: a 36-year-old Nigerien or Nigerien national (depending on the report), a storekeeper in a land transport company, who had travelled along the Lomé (Togo)–Accra (Ghana)–Abidjan (Ivory Coast)–Ouagadougou (Burkina Faso) axis [4]. After that, two other COVID-19 cases were confirmed in Niamey: a Brazilian woman, who returned to Niger on 16 March 2020, coming from Switzerland, and a 51-year-old Italian man, who returned to Niger on 28 February 2020, from his home country of Italy [3]. On 24 March, the first death from the new coronavirus in Niger was recorded: a 62-year-old male, Brazilian national, who entered Niger from Senegal on 27 February [4]. Niger declared a national state of emergency due to the COVID-19 pandemic on 27 March. Gatherings of more than 50 people were banned, places of worship closed, and obligatory epidemiological safety measures were instituted, including the wear of facemasks and social distancing in public places and public transport. On 2 April, the first two COVID-19 cases outside Niamey were confirmed in two residents of Maradi.

At the beginning of May 2020, a handful of Belgian military service men developed COVID-19 suspicious symptoms. The METT returned to Belgium in two waves, on 13 May and 19 May 2020. Immediately upon their arrival in Brussels, nasopharyngeal swabs and blood samples were collected from all soldiers, symptomatic, and asymptomatic alike, for COVID-19 diagnostic testing. The returning military personnel were placed in quarantine nearby the military airport, awaiting test results and medical consultation later in the day. SARS-CoV-2 viral loads of the nasopharyngeal samples were determined using quantitative RT-PCR (RT-qPCR), while two immunoassays were used to assess their antibody response to the virus. A rapid COVID-19 antibody test was used for first line screening, and a more widely available and evaluated double antigen sandwich assay was performed later on frozen serum samples. Two weeks later, all individuals were re-tested. The SARS-CoV-2 genomes present in RT-qPCR positive nasopharyngeal samples were sequenced. Viral genomes were evaluated and a retrospective epidemiological study was conducted to identify possible origins and routes of spread and to discuss measures to prevent or control future COVID-19 outbreaks, which pose a threat to the readiness of military forces and their ability to fulfill missions [5].

## 2. Materials and Methods

### 2.1. Samples for COVID-19 Diagnosis

Nasopharyngeal sample collection kits (Copan Diagnostics, Murrieta, CA, USA), consisting of a flexible minitip flocked swab in a tube filled with 1 mL Universal Transport Medium (UTM), were used for COVID-19 sample collection in all 70 returning service members, immediately upon arrival in Belgium (D0) and two weeks later (D14). Blood samples were collected, using VACUETTE serum gel tubes (Greiner Bio-One, Vilvoorde, Belgium), for COVID-19 antibody testing in all returning personnel on D0 and D14. Blood was allowed to clot for a minimum of 30 min in a vertical position and then centrifuged at room temperature in a swinging bucket rotor for 10 min at 1500 *g*. Serum samples were tested immediately and stored at −80 °C ± 5 °C for possible re-testing.

### 2.2. SARS-CoV-2 RT-qPCR

RT-qPCR was performed on the nasopharyngeal swabs using the Novel Coronavirus (2019-nCoV) Nucleic Acid Diagnostic Kit and Sample Release Reagent (both Sansure Biotech Inc., Changsha, China), according to the manufacturer’s instructions, with some adaptations for the LightCycler 2.0 Instrument (Roche Diagnostics Belgium, Machelen, Belgium). The assay targets the ORF1ab and N genes, coding for a well-conserved replicase polyprotein and the nucleocapsid protein, respectively. Each test also contains an RNA internal control (RNAse P genes), which detects RT-PCR failure and/or inhibition. Swab samples were first inactivated at 56 °C for 30 min in a bead bath. Two hundred microliters of swab medium were pipetted into a 1.5 mL tube, which was centrifuged at 13,500 *g* for 3 min. The supernatant was discarded and 500 µL of sterile saline were added. The tubes were vortexed and again centrifuged at 13,500 *g* for 3 min, and after discarding the supernatant, about 20–40 μL of the liquid were retained. Thirty microliters of Sample Release Reagent (denatures proteins and frees SARS-CoV-2 RNA) were added and mixed by pipetting up and down. The tubes were incubated at room temperature for 10 min. Eight microliters of the sample supernatants, of the negative control (physiological saline), and of the positive control (in vitro transcriptional RNA of target genes ORF1ab and N) were subsequently pipetted into the corresponding capillaries, which were preloaded with 12 μL PCR Master Mix. The program cycle parameters were as follows: a reverse transcription step at 50 °C for 30 min, followed by a cDNA pre-denaturation at 95 °C for 1 min and then 45 cycles of denaturation at 95 °C for 15 s, and annealing, extension and fluorescence collection at 60 °C for 30 s. There was no need to apply color compensation files.

### 2.3. SARS-CoV-2 Immunoassays

Two SARS-CoV-2 immunoassays were performed on the serum samples. The Multi-G COVID-19 IgG/IgM rapid test (MULTI-G, Antwerpen, Belgium), a lateral flow chromatographic membrane-based immunoassay, was used for the in vitro qualitative detection of IgG and IgM antibodies to 2019-nCoV in serum specimens. In this assay, the samples first react with SARS-CoV-2 antigen-coated particles and then migrate to react with anti-human IgG and/or IgM antibodies. According to the package insert, the relative specificity of the test is 98.0% for IgG and 96.0% for IgM. The relative sensitivity is 100% for IgG and 85.0% for IgM. The Elecsys Anti-SARS-CoV-2 immunoassay on the Cobas e 601 analyzer (Roche Diagnostics Belgium, Machelen, Belgium) was used for the in vitro qualitative detection of antibodies (including IgG) to SARS-CoV-2 in serum samples. This double antigen sandwich assay uses a mix of biotinylated and ruthenylated recombinant nucleocapsid (N) antigen of SARS-CoV-2 for determination of antibodies. A signal threshold ≥1 was defined as positive. A specificity of 99.81% and a sensitivity of 100% (≥14 days post PCR confirmation) were reported in the package insert. All immunoassays were performed according to the manufacturers’ instructions.

### 2.4. SARS-CoV-2 Genome Sequencing

D0 nasopharyngeal samples with positive RT-qPCR results were selected for sequencing on an Oxford Nanopore MinION device using R9.4 flow cells (Oxford Nanopore Technologies, Oxford, UK), based on a protocol from Quick et al., 2017 [6]. Briefly, RNA was extracted from 200 μL UTM transport medium and eluted in 80 μL water using a Maxwell RSC Instrument. 7 μL RNA was then converted to cDNA using random hexamers and the ProtoScript II First Strand cDNA Synthesis Kit (New England Biolabs, Hitchin, UK). Subsequently, a strain-specific multiplex PCR was performed in six reactions using an 800 bp SARS-CoV-2 primer scheme (Table S1) and Q5 High-Fidelity DNA polymerase (New England Biolabs). Cycling conditions were 30 s at 98 °C, followed by 25–30 cycles at 98 °C for 10 s, 65 °C for 1.5 min, 72 °C for 2 min, and a final elongation step at 72 °C for 2 min. The resulting 800 bp PCR products were pooled and cleaned up using AmpureXP magnetic beads (Beckman Coulter, High Wycombe, UK) and quantified using a Qubit dsDNA High Sensitivity assay on a Qubit 3.0 instrument (Thermo Fisher Scientific, Waltham, MA, USA). Samples were then barcoded using the Ultra II End Repair/dA-Tailing Module (New England Biolabs) and the native barcoding kits NBD104 and NBD114 (Oxford Nanopore Technologies), cleaned up with magnetic beads and pooled at equimolar ratios prior to ligation of the AMII adapters with blunt/TA ligase master mix (New England Biolabs). Sequencing libraries were loaded onto the R9.4 flow cell using the ligation sequencing kit LSK109 (Oxford Nanopore Technologies) and sequencing data were collected overnight. Sequence reads were base called in high accuracy mode and demultiplexed using the Guppy algorithm v3.6 (Oxford Nanopore Technologies). Consensus genome sequences were produced by comparing two distinct bioinformatics pipelines. First, an in-house customized pipeline, which aligns reads to a SARS-CoV-2 reference genome (GenBank MN908947.3) using Minimap2, was applied. After removal of primer sequences using a custom Python script, a majority rule consensus was produced for positions with ≥100x genome coverage, while regions with lower coverage, were masked with N characters [7]. In parallel, the Artic network’s bioinformatics pipeline [8] was run, which incorporates base quality data as well as the nanopolish algorithm [9] to improve consensus sequence for a draft genome assembly.

### 2.5. Genomic Epidemiology

Whole Genome sequences were compared to the reference SARS-CoV-2 Wuhan genome (NC_045512.2) and to all SARS-CoV-2 genomes submitted to the GISAID (Global Initiative on Sharing All Influenza Data) repository (https://www.gisaid.org) [10] (July 24 status) and to the National Center for Biotechnology Information (NCBI). BLOSUM62 substitution scores were calculated to assess amino acid changes and their potential to alter or change the functionality of the involved protein [11]. Negative scores are predicted to be “bad” in terms of substitution, i.e., they are not frequently observed in Nature. After submission of the obtained SARS-CoV-2 genome sequences to GISAID (Accession IDs: EPI_ISL_487433 to EPI_ISL_487436) they were automatically included into Nextstrain [12], an open-source project providing a continually updated phylogenetic analysis and visualization of the evolutionary relationships of publicly available SARS-CoV-2 genomes. This analysis includes subsampling, alignment, maximum likelihood phylogenetic inference, temporal dating of ancestral nodes, and discrete trait geographic reconstruction. Nextstrain phylogeny is rooted relative to early samples from Wuhan. Full details on bioinformatic processing can be found at [13]

### 2.6. Conventional Epidemiology

Demographics, COVID-19 suspicious symptoms and signs (cough, fever, sore throat, myalgia, headache, fatigue, anosmia, ageusia, and/or diarrhea) and dates of symptom onset were extracted from the medical files of the 70 Belgian soldiers who took part in ONN in Maradi, Niger. Information on the duration of COVID-19 compatible signs and symptoms was not systematically recorded. Possible contacts between the Belgian soldiers and externs were traced. Background COVID-19 related epidemiological data were retrieved from online local media and from the European Centre for Disease Prevention and Control (ECDC) (https://www.ecdc.europa.eu/en/geographical-distribution-2019-ncov-cases).

### 2.7. Ethics Statement

Clinical samples were routinely processed in view of diagnosis. Results were analyzed in the context of retrospective epidemiological analysis and were not deemed prospective research.

## 3. Results and Discussion

Seventy Belgian male soldiers returned from a mission in Maradi in two waves, one on 13 May and another on 19 May 2020. They had arrived in the Maradi military education and training center on 12 December 2019 and 1 February 2020, a few days before the first diagnosis of COVID-19 in Belgium, in a 54-year-old man, who was repatriated from Wuhan City on 3 February 2020 [3]. Mild symptoms indicative of COVID-19 were recorded in 22 (31.4%, 22/70) of these soldiers in the 3 week period (30 April to 19 May) preceding their return to Belgium (Table 1 and Table 2). During that same period, approximately one sixth of the 150 Nigerien military trainees ran a fever.

The Belgian soldiers were immediately tested (RT-qPCR and two immunoassays), nearby the military airport, upon their return to Belgium (D0) and again two weeks later (D14). Nine Belgian soldiers (12.9%, 9/70) had at least one positive COVID-19 diagnostic test result (Table 2). Five of them (55.6%, 5/9) were trainers. The total number of trainers involved in the training mission was 12 (17.1%, 12/70). Trainers were thus disproportionately represented in the COVID-19 positive diagnosis population.

SARS-CoV-2 viral load was detected in the D0 nasopharyngeal samples of four (5.7%, 4/70) soldiers (M4-M7), which persisted at D14 in three of them (Table 2). The four RT-qPCR positive soldiers remained in isolation for two weeks. In these four D0 RT-qPCR positive soldiers, antibodies were not observed at D0, but all had seroconverted on D14 (Table 2). This indicates that these soldiers were recently infected and that the Maradi outbreak was still ongoing. The remaining five soldiers with negative RT-qPCR results, showed positive immunoassays at D0, which highlights the importance of serological testing to achieve more accurate estimates of the extent of a COVID-19 outbreak. However, for two individuals (M8 and M9), only the rapid antibody tests were positive, making it uncertain whether they actually had the disease (Table 2). Seroconversion is usually, and depending on the applied method, observed after a median of approximately two weeks after symptom onset for both IgM and IgG [14] and in contrast to IgG, the positive rate of IgM drops after 5 weeks [15]. Although seroconversion as well as the loss of IgM can clearly be observed in our antibody data, the timings do not always correspond. For example, in some patients IgMs disappeared more rapidly (e.g., M2 and M3) or were not detected at all (e.g., M7), but this was somewhat expected given person-related variations as well as the sensitivity of the different tests used (Table 2).

Of interest, four (M6–M9) of the nine COVID-19 positive soldiers (including soldiers M8 and M9 with doubtful diagnosis) were asymptomatic (Table 2). In two of them, SARS-CoV-2 viral load was detected by RT-qPCR (Table 2). These infected asymptomatic soldiers (one of them a trainer) would have continued to work while shedding the virus. In addition, for two soldiers (M4 and M5), the onset of COVID-19 symptoms occurred on May 15 and 16, respectively, a few days before their return to Belgium. Hence, it is very likely that the timely return to Belgium prevented this becoming worse.

For comparison, a COVID-19 outbreak on the USS Theodore Roosevelt was characterized by widespread transmission with mild symptoms and asymptomatic infection (20%) among a similar population of mostly young healthy adults living in a congregate environment [16]. Of note, just like with the Belgian soldiers, anosmia and ageusia were the symptoms most strongly associated with infection, followed by fever [16]. Some scientists suggested that onset of anosmia should be considered SARS-CoV-2 related until proven otherwise and recommended immediate isolation and confirmatory testing [17].

To analyze possible patterns of spread across local and global epidemiological scales, we further determined the whole-genome sequences of the SARS-CoV-2 isolates from the four D0 RT-qPCR positive Belgian soldiers (sequencing statistic are given in Table S2). The genome sequences were submitted to GISAID under accession IDs EPI_ISL_487433-6, and were re-named C2181, C2182, C2183, and C2185 in Nextstrain (Table 3). No other SARS-CoV-2 genome sequences from patients with documented history of exposure or residence in Niger were present in the GISAID and NCBI databases. The four genome sequences were compared to the reference SARS-CoV-2 Wuhan genome (NC_045512.2) and with all SARS-CoV-2 genomes submitted to the GISAID and NCBI repositories. Genomes C2181 and C2185 are identical and were found in soldiers M4 and M7, both trainers (Table 3). Genomic sequences of samples collected from COVID-19 cases within one infection cluster are often identical or highly similar, especially within household pairs [18]. However, due to the relatively limited mutation rate of SARS-CoV-2, it is impossible to know if both trainers were infected by the same source, by different sources, or passed it on to each other. Genome C2183 has one additional difference in comparison to genomes C2181 and C2185, i.e., mutation G20477T corresponding to a glycine (G) to valine (V) amino acid change in protein ORF1ab. Genome C2182 has two differences in comparison to genomes C2181 and C2185: mutations A8095C and C19164T, both in ORF1ab and both silent (Table 3).

With two genomes being indistinguishable and one differing by one single-nucleotide polymorphism (SNP), and another by two SNPs, the genomic variations observed in our cluster correspond with those observed in “institutional outbreaks”. In three reported outbreaks where 12, 17, and 35 individuals had contracted the virus within one institution, up to two SNPs were detected [18].

**Figure 1:**
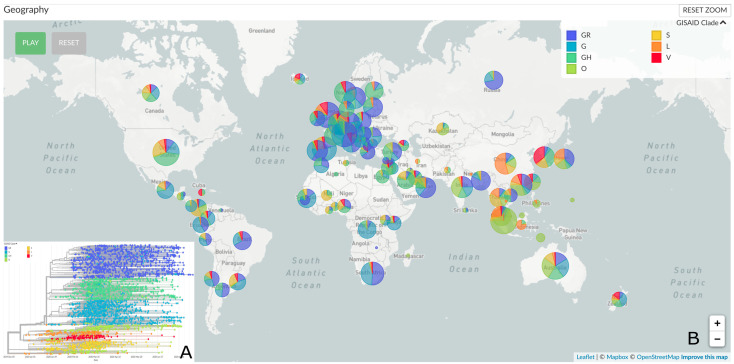
(**A**) Global phylogenetic analysis of SARS-CoV-2. The time-scaled tree was generated by the Nextstrain open source project showing genetic divergence of 4311 SARS-CoV-2 genomes submitted between December 2019 and August 2020 (accessed 11 August 2020). (**B**) Global distribution of the different SARS-CoV-2 clades defined by GISAID (i.e., GR, G, GH, O, S, L, and V).

**Figure 2:**
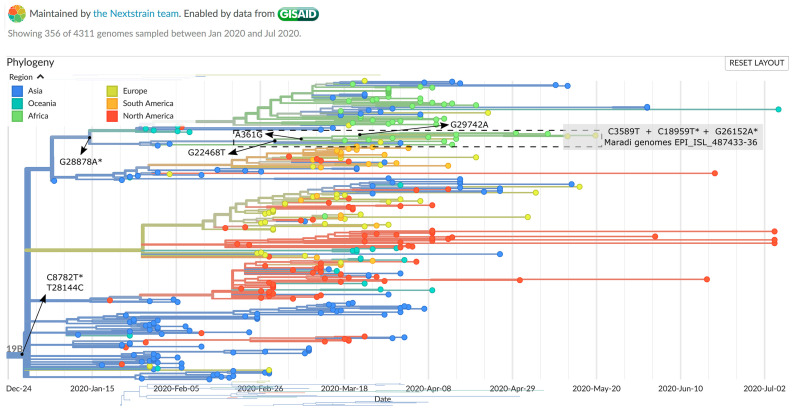
Nextstrain phylogenetic analysis (accessed 11 August 2020) focusing on the genetic divergence of 356 SARS-CoV-2 genomes belonging to the S/19B clade. Branch colors represent the estimated region of exposure. Terminal node colors represent the region of submission. Arrows indicate the mutations that occurred at ancestral nodes. Mutations that result in an amino acid change are denoted by an asterix. The grey box indicates the placement of the Maradi genomes. Only two of the four Maradi genomes are shown (Belgium/ITM_C2182 and ITM_C2183) as Nextstrain selects a subsample for visualization. The section defined by the dashed rectangle is enlarged in Figure 3.

**Figure 3:**
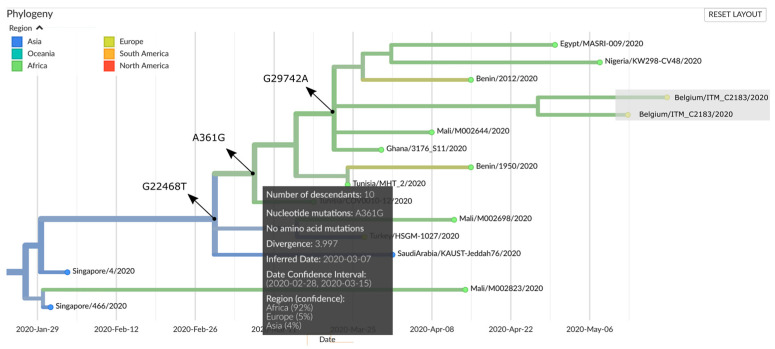
Rectangular sub-tree generated by Nextstrain (accessed 11 August 2020) showing the A361G defined subcluster harboring the Maradi SARS-CoV-2 genomes. Arrows indicate the mutations that occurred at ancestral nodes. Branch colors represent the estimated country of exposure. Terminal node colors represent the country of submission. Nextstrain selected only two of the four Maradi genomes (Belgium/ITM_C2182 and ITM_C2183) for visualization, designated by the grey box.

To date, five major clades of SARS-CoV-2 were defined (i.e., GR, G, GH, L, O, S, and V), based on characteristic mutational events observed in the genomes submitted to GISAID (https://www.gisaid.org/epiflu-applications/next-hcov-19-app/) (Figure 1) [19]. Phylogenetic examination using Nextstrain (accessed 11 August 2020) showed that all four Maradi genomes belonged to cluster 19B of GISAID’s clade S (Figure 2). The S clade was first observed among travelers from Wuhan in the early days of the outbreak [20] and was prevalent in North America and in Europe [19,20,21], but has now declined worldwide in favor of the G clades (Figure 1B). All four Maradi genomes bear the two signature mutations of clade S: the silent mutation C8782T and mutation T28144C, which causes a leucine (L) to serine (S) (hence the clade’s name) change in the ORF8 protein [18]. In addition, our cluster shared 7 other mutations when compared to the reference NC_045512.2, four of which were previously reported and determine the tree topology (G28878A, G22468T, A361G, and G29742A), as indicated by the arrows in Figure 2 and Figure 3. Mutation G26152A, which causes a glycine (G) to arginine (R) amino acid change in ORF3a, and the silent mutation C3589T were previously observed in other SARS-CoV-2 genomes. The C18959T mutation, however, is unique to the Maradi genomes and results in an alanine (A) to valine (V) amino acid change in ORF1ab. Other infrequent variations, which are present in less than 10 reported genomes, are shown in Table 4.

The rare silent mutation A361G, present in all four Maradi genomes, was recently observed in Benin, Nigeria, and Mali (Table 4), three neighboring countries of Niger, and places our genomes in a cluster of recent African descent (92% confidence), with its most recent common ancestor (MRCA) estimated between 28 February and 15 March 2020 (Figure 3). This is compatible with the suspected first COVID-19 case in Niger (reported on 19 March 2020), who had travelled to Togo, Ghana, Ivory Coast, and Burkina Faso [3]. The dates on which the first confirmed COVID-19 cases were reported in these countries, according to ECDC, were 7 (1 case), 13 (2 cases), 12 (1 case), and 11 March 2020 (2 cases), respectively.

Three amino acid changes, resulting from the individual mutation G20477T, the shared mutation G26152A, and the S clade signature mutation T28144C are predicted to result in significant alterations of the functionality of the involved proteins (negative BLOSUM62 scores) (Table 3) [10]. The mutation predicted to have the most significant impact (exhibiting a BLOSUM score of −3), was G20477T, but has only been observed once so far, in March 2020, in isolate nCoV-19/France/10063BI/2020 (Table 4). It was suggested that SARS-CoV-2 protein sequence diversity could be associated with virus pathogenicity and/or transmission. In Europe, several countries were associated with specific virus clades or mutations while the frequency in clinical symptoms of COVID-19 patients also varied between these countries. It was therefore suggested that genome and patient symptom data should be combined to better understand SARS-CoV-2 pathogenicity and help develop adapted treatments [20].

A military camp is a semi-enclosed environment with a high population density and contained spaces (e.g., dining halls and recreational spaces) in which many soldiers (e.g., trainers and trainees) and a number of civilians interact. Communal living and training environments are renowned for person-to-person transmission of disease agents, a frequent problem for militaries worldwide [4]. During their entire stay at the Maradi training facility, the 70 Belgian military service men had sporadic contacts with Nigerien civilians (in the camp and in the city of Maradi), but especially the trainers had frequent contacts with 150 Nigerien military trainees (Table 5).

**Figure 4:**
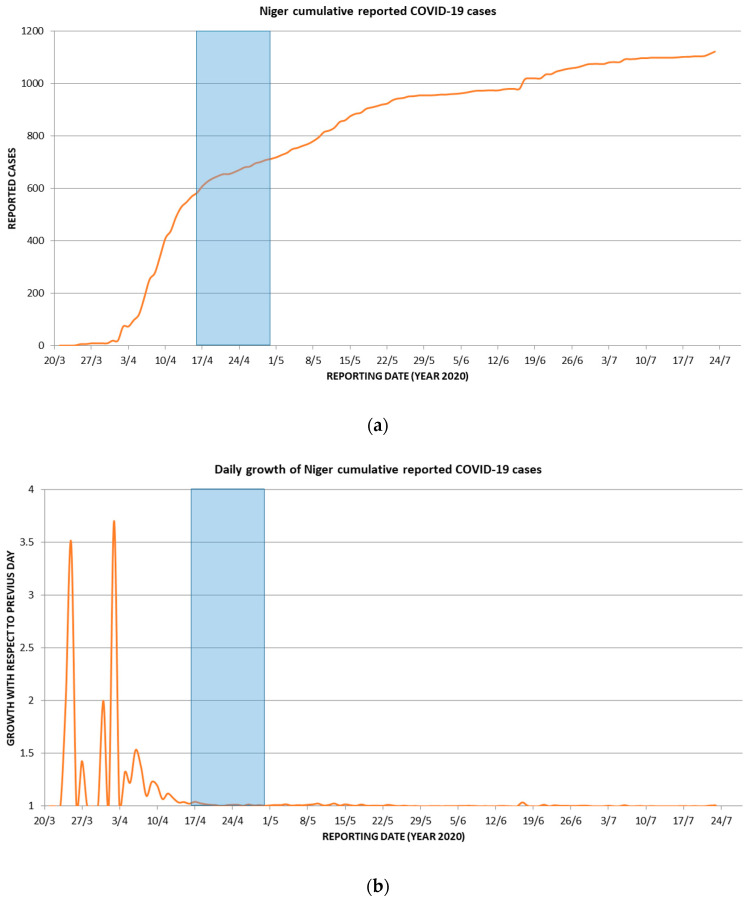
Window of possible COVID-19 infection for the Belgian soldiers in Maradi plotted (blue bars) on a background of charted ECDC data on the reported COVID-19 cases in Niger (red curves): (**a**) cumulative reported COVID-19 cases; (**b**) daily growth of cumulative reported COVID-19 cases.

Other non-Nigerien contacts occurred at least two weeks before the date on which the first COVID-19 cases were presumed to have appeared in Maradi (2 April) and more than six weeks before the onset of COVID-19-like symptoms in the Belgian military service men (30 April–16 May) (Table 1 and Table 2). Considering that the incubation period for COVID-19 is currently estimated to be between one and 14 days [22], it is unlikely that the Belgian soldiers contracted COVID-19 from these non-Nigerien contacts. In fact, the possible window of COVID-19 infection of the Belgian soldiers occurred short after high daily growth increases of COVID-19 cases in Niger (more than doubling for some days) (Figure 4), as derived from data reported by the ECDC (https://www.ecdc.europa.eu/en/geographical-distribution-2019-ncov-cases).

Overall, the data produced by this combined conventional (i.e., contact tracing) and genomic (i.e., African MRCA) epidemiological analysis, suggest that the Belgian soldiers were infected via direct contacts with locals and subsequently among themselves.

## 4. Conclusions

The SARS-CoV-2 outbreak in a Belgian military education and training center in Maradi, Niger, was characterized by mild symptoms in five soldiers and asymptomatic infection in two soldiers (one trainer), both having a viral load, as diagnosed upon their timely return to Belgium. The symptoms most strongly associated with COVID-19 infection were anosmia and ageusia. Trainers were disproportionately infected. Conventional and genomic epidemiological data suggest that the Belgian military servicemen were infected by a locally transmitted virus with a recent African common ancestor.

Based, among others, on this epidemiological study, the medical military command decided to continue testing Belgian soldiers for SARS-CoV-2 viral load and antibodies, and this two to three days before a mission abroad or on the high seas, and for specific missions immediately upon their return. Soldiers in whom SARS-CoV-2 viral load is detected are isolated. Some military operational settings (e.g., training camps in austere environments and ships) were also equipped with mobile infectious disease (COVID-19) testing capacity. More specifically, the training center in Maradi is now using a GeneXpert machine for the rapid detection of SARS-CoV-2 viral load in nasopharyngeal swabs.

## Figures and Tables

**Table 1:** Symptoms and signs indicative of COVID-19 in Belgian military returning from Maradi (*n* = 22).

Symptoms	COVID-19 Diagnosis	
	At Least One Positive Test (*n* = 5)	No Positive Tests (*n* = 17)
Anosmia/ageusia	4	0
Diarrhea	1	6
Sore throat/blocked nose	2	8
Cough	0	4
Myalgia	2	5
Post exercise fatigue	2	5
Fever	3	1
Headache	2	6

**Table 2:** Demographics, symptomatology, and test results of Belgian military service men with positive COVID-19 RT-qPCR and/or serology.

ID	Demographics				Symptomatology			COVID-19 Diagnostic Test Results									
	Gender (M/F)	Age (Years)	Function	Stay in the Maradi Training Center	Symptoms (Y/N)	Date Onset of Symptoms	Symptoms ^1^	Viral Load (Ct)				Antibodies					
								D0		D14		D0			D14		
								N gene	ORF 1ab	N gene	ORF 1ab	IgG ^2^	IgM ^2^	Elecsys ^3^ (Signal)	IgG ^2^	IgM ^2^	Elecsys ^3^ (Signal)
M1	M	32	Trainer	Feb 1–May 19	Y	30 April	AA, D, F	Neg	Neg	Neg	Neg	**POS**	Neg	Neg (0.405)	**POS**	Neg	**POS (5.25)**
M2	M	23	Trainer	Feb 1–May 19	Y	3 May	STBN, M, PEF, F	Neg	Neg	Neg	Neg	Neg	**POS**	Neg (0.135)	**POS**	Neg	**POS (3.42)**
M3	M	26	Trainer	Feb 1–May 19	Y	6 May	AA, M, F, H	Neg	Neg	Neg	Neg	**POS**	**POS**	Neg (0.271)	**POS**	Neg	**POS (2.67)**
M4	M	29	Trainer	Feb 1–May 19	Y	15 May	AA, STBN, PEF, H	**POS (28.9)**	**POS (29.8)**	Neg	Neg	Neg	Neg	Neg (0.091)	**POS**	Neg	**POS (2.33)**
M5	M	28	JSD	Feb 1–May 19	Y	16 May	AA	**POS (24.5)**	**POS (25.0)**	**POS (36.9)**	Neg	Neg	Neg	Neg (0.94)	**POS**	POS	**POS (1.57)**
M6	M	45	JSD	Dec 12–May 13	N	NA	NA	**POS (26.1)**	**POS (26.9)**	**POS (36.6)**	**POS (36.8)**	Neg	Neg	Neg (0.089)	**POS**	POS	**POS (8.14)**
M7	M	23	Trainer	Feb 1–May 19	N	NA	NA	**POS (34.1)**	**POS (34.5)**	**POS (35.6)**	**POS (36.2)**	Neg	Neg	Neg (0.106)	**POS**	Neg	**POS (1.23)**
M8	M	44	Medic	Feb 1–May 19	N	NA	NA	Neg	Neg	Neg	Neg	**POS**	**POS**	Neg (0.09)	**POS**	Neg	Neg (0.086)
M9	M	25	FP	Dec 12–May 13	N	NA	NA	Neg	Neg	Neg	Neg	**POS**	Neg	Neg (0.087)	Neg	Neg	Neg (0.086)

^1^ AA, Anosmia/ageusia; D, Diarrhea; F, Fever; H, Headache; M, Myalgia; NA, Not applicable; PEF, Post exercise fatigue; STBN, Sore throat/blocked nose. ^2^ Multi-G COVID-19 IgG/IgM rapid test. ^3^ Elecsys Anti-SARS-CoV-2 assay. A signal threshold ≥1 was defined as positive. Ct, cycle threshold; FP, Force Protection; JSD, Joint Support Detachment. The nine soldiers were ordered in the probable order of infection, based on the date of onset of symptoms, viral load and serological profile. Positive results are shown in bold. Only mild COVID-19 symptoms were observed in the positive diagnosis group, with anosmia/ageusia (4/5, 80%) and fever (3/5, 60%) being the most frequent, in contrast to the negative diagnosis group, where these olfactory/taste disorders and fever were observed in none (0%) and one (5.8%) of the 17 representatives, respectively (Table 1). None of the soldiers needed to be hospitalized. This was in the line of expectations, as these active duty soldiers were mostly relatively young (mean age of 32.3 years, range: 21–54 years), in a state of good physical fitness and had no chronic medical comorbidities and would thus have been less susceptible to infection and associated complications than the general population.

**Table 3:** Nucleotide and amino acid comparison of the 4 Maradi genomes to the reference SARS-CoV-2 Wuhan genome (NC_045512.2). Reference genome nucleotides and amino acids showing variations in any of the Maradi genomes are presented in dark green. Matches with the reference genome are indicated in light green, variations in red. The SARSCoV-2 genes harboring these variations are marked in orange, sky blue, air force blue, greige, purple and grey.

Genome Name	Patient ID	Nucleotide Variations (Positions)											
		361	3589	8095	8782	18,959	19,164	20,477	22,468	26,152	28,144	28,878	29,742
**Reference (NC_045512.2)**		A	C	A	C	C	C	G	G	G	T	G	G
ITM C2181 (EPI_ISL_487433)	M4	G	T	A	T	T	C	G	T	A	C	A	A
ITM C2185 (EPI_ISL_487436)	M7	G	T	A	T	T	C	G	T	A	C	A	A
ITM C2183 (EPI_ISL_487435)	M6	G	T	A	T	T	C	T	T	A	C	A	A
ITM C2182 (EPI_ISL_487434)	M5	G	T	C	T	T	T	G	T	A	C	A	A
		**Amino Acid (AA) Variations**											
**SARS-CoV-2 Involved Genes**		ORF1ab							S (Spike)	ORF3a	ORF8	N	3′UTR
Reference (NC_045512.2)						A		G		G	L	S	
ITM C2181 (EPI_ISL_487433)	M4	Silent	Silent		Silent	V			Silent	R	S	N	Untranslated
ITM C2185 (EPI_ISL_487436)	M7	Silent	Silent		Silent	V			Silent	R	S	N	
ITM C2183 (EPI_ISL_487435)	M6	Silent	Silent		Silent	V		V	Silent	R	S	N	
ITM C2182 (EPI_ISL_487434)	M5	Silent	Silent	Silent	Silent	V	Silent		Silent	R	S	N	
		**Weight of AA Variations (BLOSUM62 Matrix)**											
**AA Variation**						A/V		G/V		G/R	L/S	S/N	
Weight						0		−3		−2	−2	1	
Clade signature mutations					S						S		

**Table 4:** Hits for infrequent variations in the four Maradi SARS-CoV-2 genomes in the GISAID and NCBI databases (status July 24).

Variation	Genome Name	GISAID ID	Collection Date
A361G, shared by all four genomes	hCoV-19/USA/VA_6171/2020	EPI_ISL_424907	06/03/2020
	hCoV-19/USA/VA-DCLS-0021/2020	EPI_ISL_419713	11/03/2020
	hCoV-19/Mali/M002644/2020	EPI_ISL_487450	08/04/2020
	hCoV-19/Benin/2012/2020	EPI_ISL_476831	15/04/2020
	hCoV-19/Egypt/MASRI-009/2020	EPI_ISL_483035	30/04/2020
	hCoV-19/Egypt/MASRI-3/2020	EPI_ISL_475746	May 2020
	hCoV-19/Nigeria/KW298-CV48/2020	EPI_ISL_487107	08/05/2020
C3589T, shared by all four genomes	SARS-CoV-2/human/USA/VA-DCLS-0543/2020	EPI_ISL_485844	12/03/2020
	SARS-CoV-2/human/USA/VA-DCLS-0002/2020	EPI_ISL_418956	17/03/2020
	SARS-CoV-2/human/USA/VA-DCLS-0003/2020	EPI_ISL_418957	17/03/2020
	SARS-CoV-2/human/USA/VA-DCLS-0004/2020	EPI_ISL_418958	14/03/2020
	SARS-CoV-2/human/USA/UNC_200323/2020	Not applicable	April 2020
	SARS-CoV-2/human/USA/UNC_200300/2020	Not applicable	April 2020
G20477T, specific for genome C2183	hCoV-19/France/10063BI/2020	EPI_ISL_447679	March 2020
C19164T, specific for genome C2182	hCoV-19/USA/WI-GMF-M00002/2020	EPI_ISL_455574	17/05/2020
	hCoV-19/USA/WI-UW-442/2020	EPI_ISL_484817	14/06/2020

**Table 5:** Timeline and type of close contacts between the Belgian military service men and externs.

Time Period	Externs	Type of Contact
Arrival between 17 and 27 December 2019	IQARUS Damage Control Surgery Team, consisting of medics from Bulgaria (*n* = 2), France (*n* = 1), Germany (*n* = 1) and South Africa (*n* = 1)	The team stayed at the Maradi training center and shared most of the camp’s facilities with the Belgian military
4 to 5 February 2020	Visit of two Belgian senior officers	The officers stayed at the Maradi training center and shared most of the camp’s facilities with the Belgian military
Arrival between 17 February and 10 March 2020	IQARUS Damage Control Surgery Team, consisting of medics from South Africa (*n* = 2), UK (*n* = 2), and France (*n* = 1)	The team stayed at the Maradi training center and shared most of the camp’s facilities with the Belgian military
27 February 2020	Visit of USA military stationed in Niamey, Niger	Contact with Belgian medics
10 to 11 March	Visit of two Belgian senior officers	The officers stayed at the Maradi training center and shared most of the camp’s facilities with the Belgian military
During the entire stay	Nigerien military trainees	Frequent contacts with several Belgian military service men, especially with the military trainers
During the entire stay	Nigerien civilians	Several Belgian military service men had sporadic contacts with Nigerien civilians during visits to the city of Maradi

## Supplementary Materials

The following are available online at https://www.mdpi.com/1999-4915/12/9/949/s1, Table S1: SARS-CoV-2 primer scheme, Table S2: SARS-CoV-2 sequencing statistics.
